# Influence of the First Chromophore-Forming Residue on Photobleaching and Oxidative Photoconversion of EGFP and EYFP

**DOI:** 10.3390/ijms20205229

**Published:** 2019-10-22

**Authors:** Tirthendu Sen, Anastasia V. Mamontova, Anastasia V. Titelmayer, Aleksander M. Shakhov, Artyom A. Astafiev, Atanu Acharya, Konstantin A. Lukyanov, Anna I. Krylov, Alexey M. Bogdanov

**Affiliations:** 1 Department of Chemistry, University of Southern California, Los Angeles, CA 90089, USA; tirthens@usc.edu (T.S.); atanuach04@gmail.com (A.A.);; 2 Shemyakin-Ovchinnikov Institute of Bioorganic Chemistry, Moscow 117997, Russia; sphingozin@gmail.com (A.V.M.); nastia131313@gmail.com (A.V.T.); k.lukyanov@skoltech.ru (K.A.L.); 3 Semenov Federal Research Center for Chemical Physics, Moscow 119991, Russia; physics2007@yandex.ru (A.M.S.); astafiev.artyom@gmail.com (A.A.A.); 4 Moscow Institute of Physics and Technology, Dolgoprudny, Moscow 141701, Russia; 5 Present address: School of Physics, Georgia Institute of Technology, Atlanta, GA 30332, USA; 6 Center of Life Sciences, Skolkovo Institute of Science and Technology, Moscow 121205, Russia

**Keywords:** fluorescent proteins, GFP, quantum mechanics/molecular mechanics (QM/MM), atomistic calculations, photostability, fluorescence spectroscopy, light-induced oxidation, chromophore, redding, excited-state lifetime

## Abstract

Enhanced green fluorescent protein (EGFP)—one of the most widely applied genetically encoded fluorescent probes—carries the threonine-tyrosine-glycine (TYG) chromophore. EGFP efficiently undergoes green-to-red oxidative photoconversion (“redding”) with electron acceptors. Enhanced yellow fluorescent protein (EYFP), a close EGFP homologue (five amino acid substitutions), has a glycine-tyrosine-glycine (GYG) chromophore and is much less susceptible to redding, requiring halide ions in addition to the oxidants. In this contribution we aim to clarify the role of the first chromophore-forming amino acid in photoinduced behavior of these fluorescent proteins. To that end, we compared photobleaching and redding kinetics of EGFP, EYFP, and their mutants with reciprocally substituted chromophore residues, EGFP-T65G and EYFP-G65T. Measurements showed that T65G mutation significantly increases EGFP photostability and inhibits its excited-state oxidation efficiency. Remarkably, while EYFP-G65T demonstrated highly increased spectral sensitivity to chloride, it is also able to undergo redding chloride-independently. Atomistic calculations reveal that the GYG chromophore has an increased flexibility, which facilitates radiationless relaxation leading to the reduced fluorescence quantum yield in the T65G mutant. The GYG chromophore also has larger oscillator strength as compared to TYG, which leads to a shorter radiative lifetime (i.e., a faster rate of fluorescence). The faster fluorescence rate partially compensates for the loss of quantum efficiency due to radiationless relaxation. The shorter excited-state lifetime of the GYG chromophore is responsible for its increased photostability and resistance to redding. In EYFP and EYFP-G65T, the chromophore is stabilized by π-stacking with Tyr203, which suppresses its twisting motions relative to EGFP.

## 1. Introduction

Fluorescent proteins (FPs) constitute a unique group of the genetically encoded fluorescence probes with the chromophore formed from their own amino acid residues. Genetic encodability and self-sufficient chromophore maturation determine the high value of FPs as the multipurpose imaging tools. Protein engineering has played an essential role in the development of the available FPs palette, which currently includes dozens of spectral variants. Introduction of only two mutations (F64L and S65T) to the first described wild-type FP—avGFP—has resulted in enhanced green fluorescent protein (EGFP) [1], the most popular fluorescent protein yet. In EGFP, the chromophore dwells almost exclusively in the bright anionic state (fluoresces at λ_ex_ = 490 nm/2.53 eV, λ_em_ = 510 nm/2.43 eV), whereas the wild-type avGFP chromophore exists mostly in the protonated form (λ_abs_ = 395 nm/3.14 eV) and is weakly fluorescent. One more representative of the classic FPs, enhanced yellow fluorescent protein (EYFP), in which Ser65Gly/Thr203Tyr substitutions lead to 35 nm fluorescence excitation bathochromic shift relative to avGFP, was also derived from avGFP [2].

The bicyclic chromophore of avGFP and most of its derivatives (including EGFP and EYFP) is formed from the -X65-Tyr66-Gly67- tripeptide motif by autocatalytic post-translational modification that involves consecutive cyclization, dehydration, and oxidation [3,4,5]. Tyr66 and Gly66 are highly conserved in the native FPs.

Despite the variability of the first residue in a chromophore triad (X), proteins with different amino acids in this position form very similar chromophores. Thus, a typical GFP-like chromophore can be found in avGFP with SYG triad, EGFP with TYG, and EYFP with GYG. Although amino acid at the first position only weakly affects the structure of the chromophore core, it is essential for the interaction of the chromophore with its protein environment which, in turn, dramatically influences properties of the fluorescent protein, in particular those relevant for applications, such as fluorescence brightness and lifetime, Stokes shifts, and photostability [6,7,8]. The importance of the 65th position can be illustrated by the fact that Ser65 substitution by Gly, Ala, Cys, Val, or Thr suppresses the shortwave neutral chromophore absorbance peak (395 nm/3.14 eV) in favor of the anionic chromophore’s peak at 470–490 nm (2.64–2.53 eV) [2,9,10]. In EGFP, the S65T mutation causes a significant rearrangement of the hydrogen-bond network in the chromophore region [2,11,12]: the threonine residue forms a new hydrogen bond with Val61 [11]. Also, Thr65 induces Glu222 protonation and accelerates chromophore maturation (maturation time constant is 0.45 h in GFP-S65T versus 2 h in wild-type avGFP) speeding up the rate-limiting oxidation reaction rate [9,13]. In EYFP, S65G, and V68L substitutions result in a 0.9 Å shift of the chromophore towards the barrel surface relative to its position in GFP-S65T and wild-type avGFP [14]. Both mutations also improve brightness of the cells expressing respective mutants relative to the avGFP-expressing cells, probably due to their effect on the protein folding or chromophore maturation [1]. Remarkably, the shift of the chromophore observed in EYFP (and linked to the S65G substitution) leads to the appearance of the fluorescence sensitivity to halide and nitrate anions in this protein [15].

Mutational analysis of the first chromophore-forming amino acid position (Ser65 in avGFP) had been carried out in several studies and aimed primarily at determining the influence of this position on the maturation of the chromophore [13] and its basic spectral characteristics [2]. However, a systematic analysis of the influence of this position on the less obvious physicochemical characteristics of fluorescent proteins, such as photostability, fluorescence lifetime, blinking, and excited-state reactions, mediated by the molecular interactions of the chromophore with the nearest protein environment, to the best of our knowledge, has not yet been carried out. Existing data indicate that the amino acid in the 65th position has a significant impact on the GFP photophysics. For example, the EGFP mutants carrying the T65G substitution show significantly reduced quantum yield, shorter fluorescence lifetime, and an increased extinction coefficient [16,17]; an increased photostability of such proteins was also reported [17]. At the same time, in EYFP carrying the same GYG chromophore, the quantum yield is even higher and the lifetime is longer than EGFP with the TYG chromophore. 

The effect of the 65th position on the ability of GFP-like proteins to undergo light-induced oxidative green-to-red photoconversion (called oxidative redding) is also of interest. Redding was described for green proteins of different taxonomic origin with Thr, Ser, Cys, Asn, Lys, and Gly in the first position of the chromophore, but the efficiency of the red spectral form appearance was maximal in EGFP (Thr65) [18]. As for EYFP, it is capable of redding only in the presence of halide anions, and even if they are present, it is much less effective than in EGFP [19]. The current mechanistic hypothesis [19] states that the redding is initiated by the electron transfer from the electronically excited chromophore to a nearby residue. Consequently, the effectiveness of this gateway step determines the ultimate yield of the red form. Under the same hypothesis, the yield of bleaching is also correlated with the effectiveness of the photoinduced electron transfer. The calculations of the energetics of one-electron oxidation and possible electron transfer pathways suggested that excited-state electron transfer proceeds through a hopping mechanism via Tyr145; the role of Tyr145 in redding has been confirmed by mutagenesis [19]. In YFPs, the π-stacking of the chromophore with Tyr203 reduces its electron-donating ability, which can be restored by halide binding, due to its effect on the π-stacking [19]. However, a possible role of Gly65 was not investigated.

In this contribution, we examine the mutants of EGFP and EYFP proteins with reciprocal substitutions at the 65th position, EGFP-T65G and EYFP-G65T, focusing on their brightness, photostability, fluorescence lifetime, and redding ability compared to parental proteins. To rationalize the observed differences, we carried out quantum chemical and molecular dynamics simulations to estimate radiative and radiationless decay rates. On the basis of these calculations, we developed a kinetic model of the photocycle, which provides a unified picture of how the chromophore’s structure affects the photophysical properties of fluorescent proteins. The simulations revealed that the main effect of the T65G mutation is the reduced excited-state lifetime of the GYG chromophore, resulting in its increased photostability. The effect of the residue in position 65 on the brightness and quantum yield is explained by an interplay between the radiative and radiationless relaxation channels. The effect of mutation 65 in EYFP is modulated by the π-stacking interactions between the chromophore and Tyr203.

## 2. Results

### 2.1. Mutants General Description (Spectral Characteristics)

**Figure 1:**
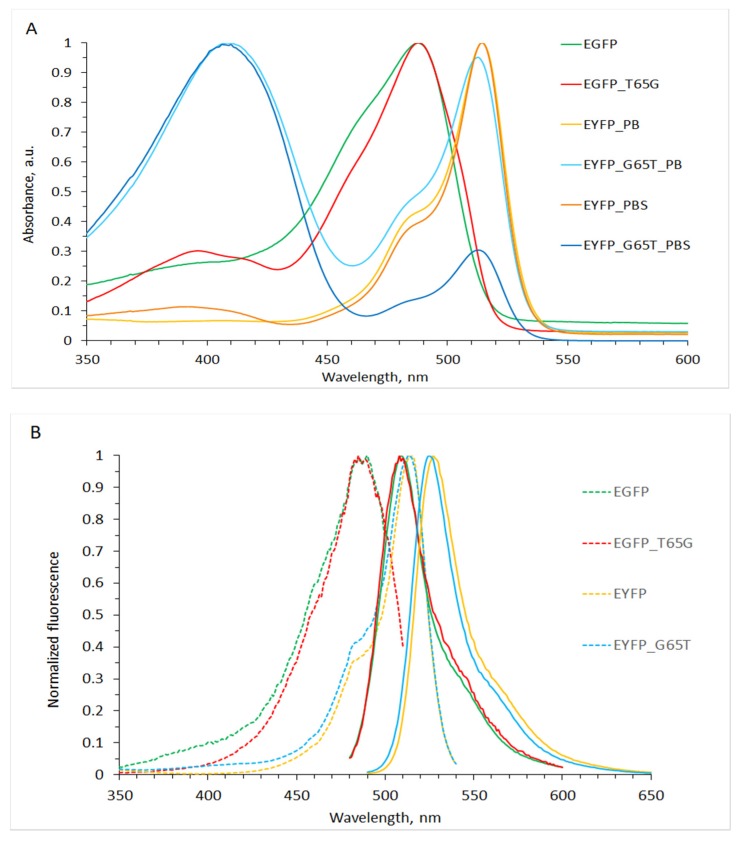
(**A**) Absorption and (**B**) fluorescence spectra of enhanced green fluorescent protein (EGFP), enhanced yellow fluorescent protein (EYFP), and mutants. In the fluorescence graph, dashed lines show fluorescence excitation, solid lines show fluorescence emission. ‘PB’ denotes phosphate buffer and ‘PBS’ denotes phosphate buffered saline containing sodium chloride (see text).

EYFP-G65T and EGFP-T65G mutants generally showed spectral similarity to their parental proteins (Figure 1; see also [17]). Like the original EGFP, EGFP-T65G has a single main absorption maximum, peaking at approximately 488 nm (2.54 eV) and corresponding to the anionic chromophore with fluorescence emission maximum at 510 nm (2.43 eV). The neutral (protonated) state of the chromophore in EGFP-T65G (absorption maximum 395 nm/3.14 eV) is minor, although it is more expressed than in EGFP; this is consistent with literature data on the role of Thr65 in maintaining the neutral state of Glu222 and the hydrogen-bond network favoring chromophore’s deprotonation. The absorption spectra of EYFP-G65T, which have two pronounced maxima—410 and 513 nm (3.02 and 2.42 eV, respectively), corresponding to the neutral and anionic chromophores—are distinctly different from both the parent protein (EYFP) and from EGFP-T65G.

A small (about 2 nm) blue shift in the anion and a significant (about 15 nm) red shift in the neutral chromophore absorption, which is unusual for proteins with the chromophore π-stacked with Tyr203 including EYFP [2], are noteworthy in the spectral comparison of EYFP-G65T with EYFP. Even more remarkable, however, is the observed dramatic dependence of the relative amplitudes of the peaks at 410 and 513 nm (3.02 and 2.42 eV) on the composition of the external environment. For example, in the hydrophosphate–dihydrophosphate buffer (PB, pH 7.4) the amplitudes’ ratio is about 1:1, while in the phosphate buffered saline (PBS, pH 7.4, ~140 mM Cl^−^) the ratio becomes approximately 3:1 in favor of the neutral chromophore. Therefore, the protonation state of the EYFP-G65T chromophore seems to exhibit an enhanced sensitivity to the electrostatic interactions with the solvated ions. This property makes it a promising candidate for the sensitive core of the ratiometric halide ion sensor.

Parental EYFP also shows spectral sensitivity to the buffer content. That is, having essentially no absorbance around 400 nm (3.10 eV) in PB, in PBS it absorbs at 395 nm (3.14 eV) (as the classic protonated GFP-chromophore), while decreasing its main absorbance at 515 nm (2.41 eV) peak by circa 6%. EYFP’s halide sensitivity is well known and is attributed to the shift of chromophore’s pKa induced by electrostatic interactions: fluorescence intensity decrease by about 40% at pH 7.0 was reported for this protein [14]. However, the contrast of the optical response to halide’s addition in the case of G65T mutant appears to be significantly higher.

### 2.2. Photostability

We measured the photostabilities of the mutants versus the parental proteins with and without electron acceptors in the media, aiming to reveal the influence of the T65G/G65T substitutions on the primary excited-state electron transfer process that is believed to result in a permanent bleaching. Also, for EYFP/EYFP-G65T we introduced an additional variable—halide presence—to photostability measurements testing its possible role in the excited-state chemistry. The photostability is quantified by the bleaching half-times (the time it takes for the fluorescence to drop by a factor of two), i.e., longer half-times correspond to more photostable proteins. 

**Figure 2:**
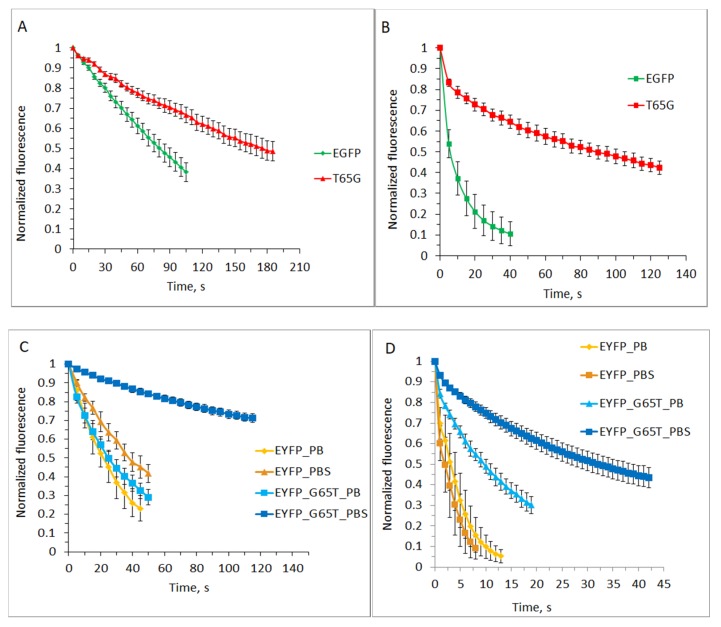
Bleaching kinetics in the immobilized proteins EGFP, EYFP, and their mutants in vitro. (**A**) Photoconversion of EGFP and EGFP-T65G in PBS. (**B**) Photoconversion of EGFP and EGFP-T65G in PBS in the presence of 0.2 mM potassium ferricyanide. (**C**) Photoconversion of EYFP and EYFP-G65T in PB and PBS (PBS contains potassium chloride). (**D**) Photoconversion of EYFP and EYFP-G65T in PB and PBS in the presence of 0.2 mM potassium ferricyanide. Green/yellow fluorescence intensities were background-subtracted and normalized to the maximum values. Standard deviation values (*n* = 15−20 measurements in a representative experiment out of five independent experiments) are shown.

The EGFP-T65G mutant (with enhanced photostability relative to EGFP in vitro and *in cellulo* [17]) showed approximately twofold higher photostability (relative photostability is defined as the ratio of the photobleaching rates) in PBS and almost 20-fold higher in PBS with 200 µM of potassium ferricyanide relative to those of EGFP (Figure 2A,B, Table 1). 

Taking into account that EGFP-T65G (EC = 70,000 M^−1^·cm^−1^; FQY = 0.06) is 8 times dimmer than EGFP, one could explain the increase of photostability in PBS by its shorter excited-state lifetime (which is also responsible for its reduced emitter efficiency). However, the degree of the photostability increase in EGFP-T65G in the presence of oxidant does not match the degree of the protein’s brightness decrease, and this probably indicates a less effective oxidative bleaching channel in this protein. The shapes of the bleaching curves with oxidant may favor this hypothesis: EGFP-T65G has a bimodal curve, but its fast component, probably related to excited-state electron transfer, is relatively short. EYFP and its mutant behavior seems more complex, in part due to the presence of an additional variable (presence or absence of the chloride anions in PBS or PB buffer, respectively) at the experimental conditions. In PB, EYFP-G65T photostability is close to that of the parental EYFP, while in PBS, the mutant shows a 5-fold decreased photobleaching rate relative to EYFP under the same conditions and around 7-fold relative to itself in PB (Figure 2C, Table 1). This observation is in accord with the absorption spectra of two proteins (Figure 1A): in the presence of 140 mM chloride only around 25% of EYFP G65T chromophore is in anionic state, which absorbs excitation light. In fact, EYFP’s photostability also somewhat increases in the presence of chloride, probably for the same reason.

Oxidant addition (200 μM potassium ferricyanide) significantly accelerates bleaching in all cases (Figure 2D, Table 1). EYFP’s photostability in PB with oxidant is reduced 6–7-fold. However, as in the case of EGFP/EGFP-T65G, the effect of ferricyanide on EYFP-G65T and EYFP behavior varies under different conditions. For EYFP with oxidant, the rate of photobleaching in PB and PBS is almost the same. One can suppose that the bleaching channel, which is dominant when electron acceptor is added, is less sensitive to chromophore’s pKa than the one that functions under normal conditions. In PB+oxidant, EYFP-G65T shows a 3-fold decrease in the photobleaching rate relative to EYFP (whereas without oxidant the rates are almost equal). In PBS+oxidant, the mutant demonstrates a 10-fold photostability increase relative to EYFP (versus 5-fold increase without oxidant), and a 3-fold increase relative to itself in PB+oxidant (versus a 6-fold increase without ferricyanide). Taken together, these ratios suggest that the oxidant reduces the dependence of the bleaching efficiency on the chromophore protonation state (see G65T_PB_ox versus G65T_PBS_ox, which changes in the presence of chloride), while the replacement of G65T generally disfavors the oxidative bleaching channel (see G65T_PB_ox versus EYFP_PB_ox).

### 2.3. Redding

We also tested redding efficiency among the mutants irradiated in presence of ferricyanide, our hypothesis being that the red form appearance rate should be inversely related to the photostability.

**Figure 3:**
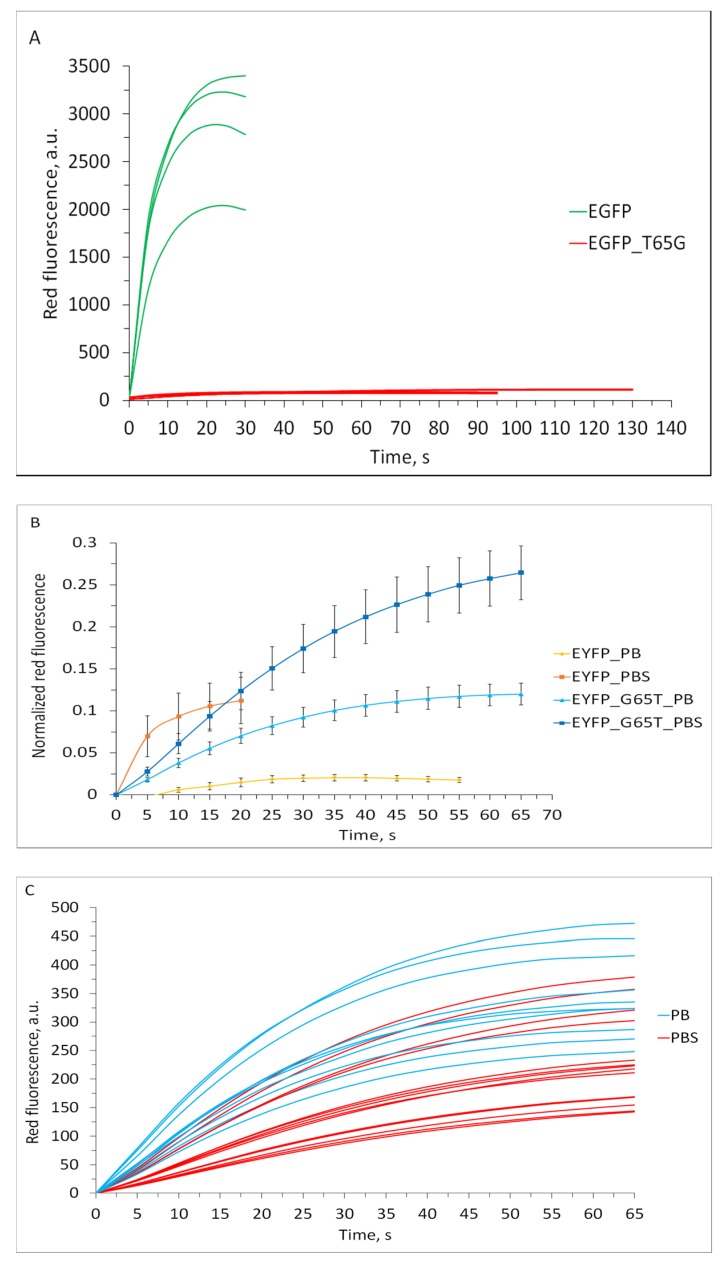
Redding kinetics in the EGFP, EYFP, and their mutants. (**A**) Appearance of red fluorescence in EGFP and EGFP-T65G. Non-normalized data for several measurements are shown. (**B**) Appearance of red fluorescence in EYFP and EYFP-G65T in PB and PBS (PBS contains potassium chloride). Averaged curves are shown. Red fluorescence intensities were background-subtracted and normalized to the maximum values. Standard deviation values (*n* = 15−20 measurements in a representative experiment out of five independent experiments) are shown. (**C**) Appearance of red fluorescence in EYFP-G65T in PB and PBS (PBS contains potassium chloride). Non-normalized data for several measurements are shown.

This appears to be true in the EGFP/EGFP-T65G pair, where the parental protein demonstrated 20–30-fold more efficient redding (and 20-fold lower photostability) (Figure 3A). As in the case of bleaching, the reduced rate of redding in EGFP-T65G cannot be explained only by the 8-fold lower relative brightness of this mutant, especially since it absorbs light even more effectively than the original protein (EC = 70,000 M^−1^·cm^−1^ versus 55,000 M^−1^·cm^−1^ in EGFP). When comparing the redding rates in different proteins, care should be taken to normalize the appearing red signal to the initial intensity of the green fluorescence. We suggest a normalization method adequate when studying redding of the same protein under slightly different conditions (for example, in cell culture). However, when comparing different proteins, this method can lead to artifacts because it does not take into account the chromophore’s ability to absorb light and its quantum efficiency.

We evaluated redding in EYFP-G65T and EYFP in PB (without chloride) and PBS (140 mM Cl^−^), both supplemented with 200 µM of ferricyanide. For EYFP in PB, we observed almost no detectable appearance of the red form (Figure 3B). We do not consider the weak growth of the red signal visible on the graph to be reliable and attribute it to the imperfectness of the procedure of the subtraction of the red component of the main spectral form leaking through the RFP filter set (see subtraction and normalization procedure in Supplementary Materials). In PBS, we detected well-expressed (both in rate and absolute value) redding, in agreement with the observations reported earlier [17]. EYFP-G65T undergoes redding both with and without chloride, demonstrating similar kinetics/rate but different yield (red signal plateau) under these two conditions (Figure 3B). It is, however, possible that the seeming quantitative difference in the redding yields of EYFP-G65T in PB and PBS represents an artifact originating from an inadequate normalization procedure. To address this issue, we also compared non-normalized datasets for EYFP-G65T redding (Figure 3C); this comparison did not confirm the trend exhibited by the normalized/averaged curves. Generally, the G65T mutation in EYFP enables the ability to undergo redding in the standard regime, i.e., independently on the halide binding. To quantitatively compare redding in PB and PBS, one should take into account an extreme sensitivity of the EYFP-G65T brightness to halide presence, which leads to a 2.5–3-fold difference in the green signal intensity at zero time. 

### 2.4. Lifetimes

Fluorescence lifetime of EGFP was measured to be 2.8 ns [13]; other studies estimated it in the range from 2.3 to 2.8 ns [17,20,21,22,23]. The spread in the reported values reflects the sensitivity towards the instrument and measurement conditions [17,20,21,22,23]. EGFP-T65G fluorescence lifetime (1.3 ns) is twice shorter than in EGFP [16].

For EYFP, fluorescence lifetime weakly depends on the halide presence (3.18 ± 0.07 ns in PB (without Cl^–^) and 3 ± 0.08 ns in PBS (with Cl^–^)), which is in rough agreement with the relevant data reported for the near homologs of EYFP [24,25]. Concurrent with our observations in the spectral domain, EYFP-G65T demonstrated a complex behavior in lifetime domain (Table 1). Thus, the mutant shows two clearly distinguishable lifetime values under 400 and 490 nm excitation wavelengths (3.10 and 2.53 eV) in the PB and PBS environment. In PB, both fluorescence decay kinetics can be fitted by the single-exponential functions with τ_400_ = 3.7 ns, τ_490_ = 4 ns. In PBS, excitation at 400 nm leads to a bi-exponential decay (τ_1_ = 3.5 ns, τ_2_ = 0.5 ns), where the faster component might be attributed to excited-state proton transfer (ESPT), although the ESPT kinetics are usually much faster than hundreds of picoseconds [26]. Excitation of the anionic form does not significantly change its fluorescent lifetime compared to the PB value.

### 2.5. Computational Results

To rationalize the observed differences in photophysical behavior of the four proteins due to the residue in position 65, we carried out the following quantum-chemical and molecular dynamics calculations:

1. Quantum-chemical calculations of the isolated model chromophores (structures, excitation energies, and oscillator strengths);
2. Molecular dynamics simulations of the model proteins in the ground and electronically excited states;
3. Hybrid QM/MM (quantum mechanics/molecular mechanics) calculations of the spectral properties of the model proteins (excitation energies and oscillator strengths for the structures taken from the ground-state molecular dynamics simulations).

**Figure 4:**
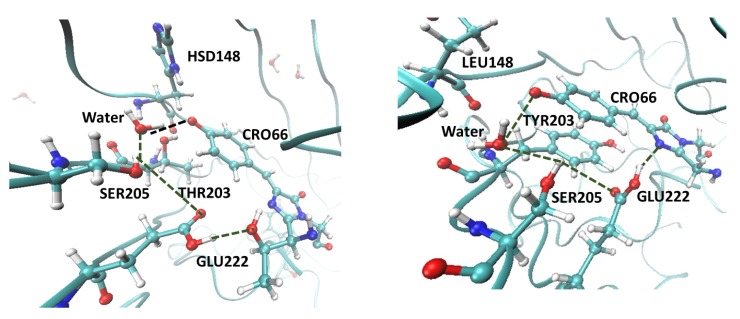
Hydrogen-bond network around the chromophore (CRO) in EGFP (**left**) and EYFP (**right**). The network includes CRO:O–water314–SER205–GLU222–CRO:O (Thr65 in EGFP). Glu222 is protonated and His148 is neutral in EGFP (protonated at δN atom). Also shown is π-stacking of the chromophore and Tyr203 in EYFP.

**Figure 5:**
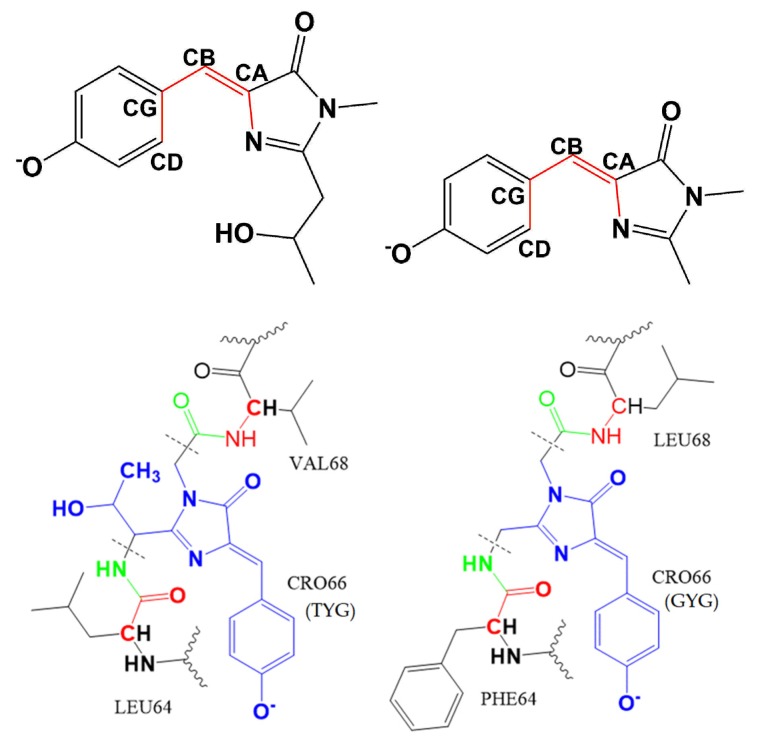
Top: Structures of the model TYG (EGFP, YFP-G65T) (**left**) and GYG (YFP, EGFP-T65G) (**right**) chromophores. Torsional angles ϕ and τ are defined as CD-CG-CB-CA and CG-CB-CA-N, respectively. The difference between the two angles Δ = ϕ − τ quantifies whether the chromophore is planar (Δ = 0) or not. Bottom: the QM/MM partitioning for EGFP (**left**) and EYFP (**right**). Blue color denotes the QM region and the black dotted lines denote the QM–MM boundary. Charges of red and green atoms were set to zero in the MM region. In EGFP-T65G, the chromophore is GYG and the neighboring residues are the same as in EGFP. Likewise, in EYFP-G65T, the chromophore is TYG and the neighboring residues are the same as in EYFP.

The results from these calculations were used to estimate radiative and radiationless lifetimes, as described below. We considered four model systems, representing EGFP, EGFP-T65G, EYFP, and EYFP-G65T. For EYFP, we carried out calculations with and without chloride anions, as in [19]. In all simulations, we considered only the deprotonated (anionic) chromophore. The protonation states of the protein residues were determined using PropKa software [27,28,29] and verified by comparing the results of the molecular dynamics simulations with the available crystal structures (2Y0G for EGFP [11], 1F0B for EYFP [15]), following the same protocols as in our earlier work [19]. Specifically, we determined that Glu222 is protonated (neutral) and His148 is neutral (HSD form, protonated at δN atom). The protonation state of Glu222 agrees with the conclusions of the experimental study [13]. These protonation states give rise to a robust hydrogen-bond network around the chromophore, as shown in Figure 4. The structures of the model chromophores and the definition of the QM/MM partitioning are shown in Figure 5. Full details of the computational protocols are provided in Section 4.6 and in the Supplementary Materials.

**Figure 6:**
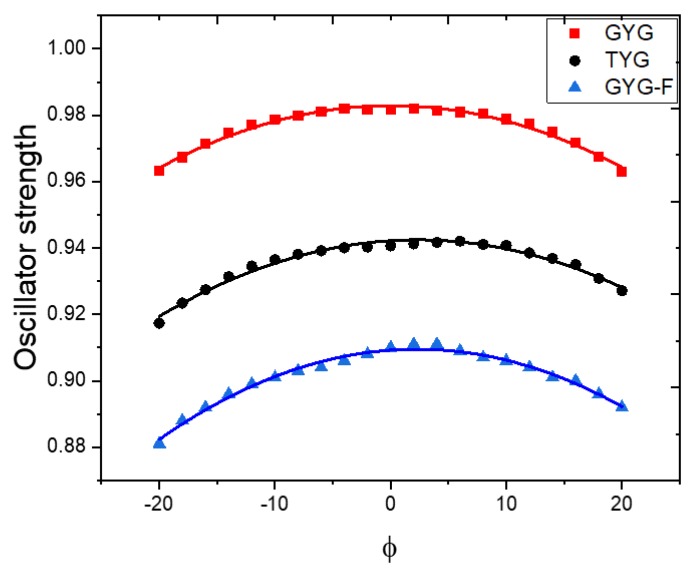
Oscillator strength for the S_0_–S_1_ transition in the isolated TYG, GYG, and fluorinated GYG (GYG-F in which one –CH_3_ is replaced with –CF_3_) chromophores along torsional angle ϕ (all other degrees of freedom are relaxed) computed with ωB97X-D/aug-cc-pVDZ.

We begin with characterization of the bare model chromophores. Figure 5 shows the structures of the isolated model chromophores and defines important structural parameters; it also shows how the chromophores are connected to the protein backbone. As one can see, the conjugated core of the TYG and GYG chromophores is the same; it comprises the phenolate and imidazolinone rings connected via the methine bridge. However, whereas the GYG chromophore is directly attached to the protein backbone through the exocyclic imidozalinone’s carbon, the TYG chromophore has an additional –CH(OH)CH_3_ tail attached to it. The presence of this tail has a relatively small effect on the excitation energy (red shift of about 0.02 eV) but leads to a 4% decrease in the oscillator strength of the bare chromophore. The –CH_3_ and –CH(OH)CH_3_ groups differ by their electron-donating ability—the presence of electronegative OH makes the latter a less effective electron donor. Thus, we attribute the larger oscillator strength in GYG relative to TYG to an increased electron density in the conjugated part of the chromophore due to stronger electron-donating ability of –CH_3_. To test this hypothesis, we carried out calculations for a fluorinated GYG chromophore in which one –CH_3_ group was replaced by –CF_3_. The fluorinated GYG chromophore shows significant reduction (7.3%) of the oscillator strength relative to the GYG chromophore, consistently with a strong electron-withdrawing ability of fluorine (see Figure 6).

As discussed below, the larger oscillator strength in GYG chromophore contributes to its increased brightness and reduced radiative lifetime (i.e., faster fluorescence). Importantly, the tail has a major effect on the hydrogen-bond network around the chromophore, its planarity and conformational flexibility. Figure 4 shows the hydrogen-bond networks around the chromophore in EGFP and EYFP. As one can see, in both proteins there are four hydrogen bonds around the chromophore. However, the Ser205–Glu222 distance (Ser205:O–Glu222:OE1) is much larger in EYFP (compare 3.74 Å in EGFP with 4.27 Å in EYFP). By allowing for larger thermal fluctuations causing transient breaking of the hydrogen bonds, a larger Ser205–Glu222 distance signifies a weaker hydrogen-bond network, which is illustrated by the results in Table 2.

Table 2 summarizes the analysis of the hydrogen-bond pattern and deviations of the chromophore from planarity in the course of the ground-state equilibrium dynamics. The average number of hydrogen bonds is smaller for the GYG chromophores relative to the proteins with TYG chromophores. Interestingly, despite a smaller number of hydrogen bonds, the deviation from planarity is smaller for EGFP-T65G relative to EGFP, both in terms of the average values of Δ and in terms of standard deviation. The latter indicates a larger dynamic range of chromophore’s motion in EGFP. A reduced range of thermal torsional motion in EGFP-T65G and smaller deviations from planarity are probably due to the bulkier size of the TYG chromophore. The EYFP chromophore shows smaller deviations from planarity, because of the stabilizing effect of the π-stacking with Tyr203 (this is consistent with the observations in [19]). The average number of hydrogen bonds around the chromophore is larger in EYFP-G65T than in EYFP because the OH group of the threonine participates in the hydrogen-bond network.

Torsional motion of the chromophore modulates the oscillator strength of the S_0_–S_1_ transition, as illustrated in Figure 6. In EGFP, the standard deviation for Δ (which quantifies the twisting motion of the chromophore) is ~17 degrees. In this range of motion the oscillator strength can be reduced by several percent (Figure 6 shows that the oscillator strength depends quadratically on ϕ). These results explain the variations in the average oscillator strengths for the S_0_–S_1_ transition for the four systems discussed below.

On the basis of the QM/MM calculations of the transition energies and oscillator strengths, we estimate intrinsic fluorescence lifetime, τ_fl_. Intrinsic radiative lifetime is inversely proportional to the oscillator strength of the transition (f_l_) and to the square of corresponding excitation energy (E_ex_). In atomic units, intrinsic radiative lifetime τ_fl_ is [30]:

(1) $$\frac{1}{\tau_{fl}}=\frac{E_{ex}^{2}f_{l}}{2\pi {\left(c^{\prime }\right)}^{3}\epsilon }$$

where *c*′ is the speed of light in the medium (*c*′ = *c*/*n*; c is the speed of light in vacuum and *n* is the index of refraction) and ϵ is the dielectric constant. For vacuum, *c* = 137 and *ϵ* = 1. Dielectric constant in proteins is small (i.e., 2–8). The index of refraction of water is 1.33; the refractivity of protein solutions is generally larger, around 1.6 [31,32].

Table 3 shows excitation energies of the isolated chromophores and average excitation energies and oscillator strengths computed for 21 QM/MM snapshots taken from the ground-state equilibrium trajectories. These values are used to compute radiative lifetimes by Equation (1) with *n* = 1 and *ϵ* = 1. The absolute values of the computed lifetimes are almost 10 times longer than the experimentally observed fluorescence lifetimes, which is expected given the uncertainties in the computed values and the key constants (i.e., *n* and *ϵ*). Using *n* = 1.6 brings the computed values down, to the range of 7–8 ns. Moreover, we note that Equation (1) provides only an upper bound of τ_fl_ and does not account for other decay channels available to such complex polyatomic systems as fluorescent proteins. However, we expect that these calculations capture the essential trend of variations in the intrinsic fluorescence lifetime due to the variations in the oscillator strength induced by thermal motions and differences in the chromophore’s structure. To zoom into this trend, the last two columns of Table 3 show relative values of the computed fluorescence lifetimes with respect to that of EGFP. The results idicate that the proteins with the GYG chromophore are expected to have intrinsic fluorescence lifetimes shorter by 8–12% than their counterparts with TYG. This difference is due to slight red shifts, dynamically reduced oscillator strengths in TYG, and the electronic effect of OH, all caused by the bulkier and more electronegative threonine group.

**Figure 7:**
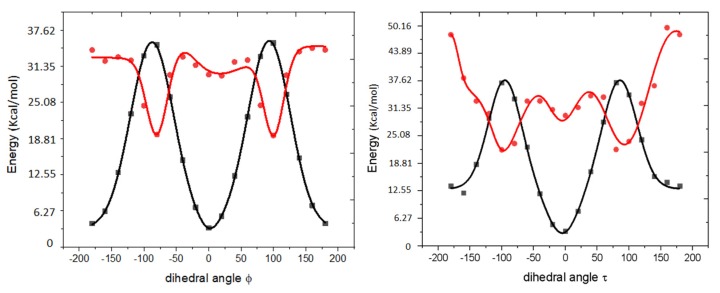
PES scans (relative energies) for the isolated GYG chromophore along the dihedral angles ϕ (**left**) and τ (**right**) in the ground (black) and electronically excited (red) states. All other degrees of freedom are frozen. The dots represent ab initio calculations (ωB97X-D/aug-cc-pvDZ) and the solid lines are fits to the force-field torsional potential used in molecular dynamics simulations (see Section 4.6). In contrast to the isolated chromophores, the protein-bound excited chromophores can only undergo phenolate flip (ϕ twist) because the imidozalinone ring is covalently bound to the protein backbone.

In contrast to a relatively modest effect of the residue in position 65 on the chromophore structure in the ground state, it has a dramatic effect on the excited-state potential energy surface, which strongly affects the dynamics of the chromophore following photoexcitation. The origin of this strong effect of hydrogen bonding is a much flatter torsional potential of the chromophore in the excited state [33,34]. Figure 7 shows the scans of the potential energy surfaces in the ground and the first excited state of the isolated GYG chromophore along the two torsional angles. As one can see, the chromophore in its ground state is rather rigid due to its π-conjugated system: the barriers for the ϕ (phenolate flip) and τ (imidozalinone flip) rotations are about 31.61 and 34.47 kcal/mol, respectively. However, in the S_1_ state (which has π–π* character), the bond order is reduced, giving rise to relatively flat potential energy profiles along the twisting coordinates (the computed barriers for the ϕ and τ rotations are 3.59 and 4.52 kcal/mol, respectively). These flat profiles are responsible for low FQY of isolated chromophores [33,34,35,36]. The hydrogen-bond network around protein-bound chromophores plays a crucial role by stabilizing the otherwise floppy structure in a planar configuration, thus preventing the chromophore’s trapping in dark twisted states and suppressing the radiationless relaxation via conical intersections. [33,34,35]. That is why different hydrogen-bond patterns around GYG and TYG chromophores have a profound effect on their excited-state dynamics. Specifically, as illustrated by excited-state molecular dynamics simulations, GYG chromophores are much more likely to twist in the excited state than the TYG chromophores.

**Figure 8:**
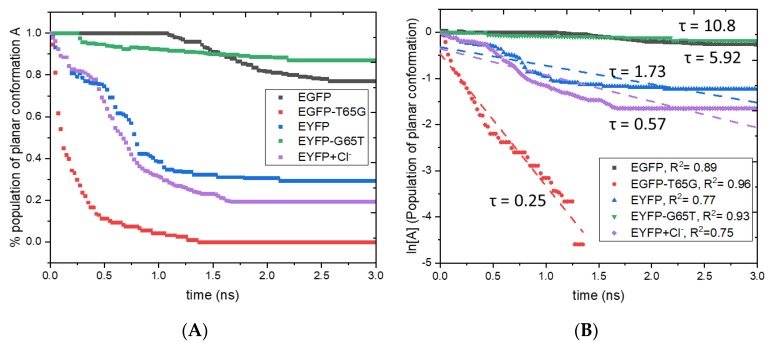
Left: Evolution of planar (**A**) population in excited-state molecular dynamics simulations of EGFP, EGFP-T65G, EYFP, EYFP-G65T, and EYFP+Cl^–^. (**B**) Right: Linear fit for ln[A].

To quantify excited-state evolution, we carried out molecular dynamics simulations using the modified force-field parameters (see Section 4.6 and Figure 7) designed for the S_1_ state. Starting from 101 snapshots harvested from the ground-state trajectories for each protein, we propagated excited-state trajectories for 3 ns; the results were saved each 2.5 ps. To estimate the rate of radiationless relaxation, we monitored the dihedral angle ϕ along simulation trajectories and defined two populations: **A** (planar chromophore, defined as ϕ < |50°|) and **B** (twisted chromophore, ϕ > |50°|). The dihedral angle τ does not fluctuate significantly (~20°) in the course of dynamics, because of the covalent bond between the imidazolinone ring and the protein’s backbone. Once the value of ϕ reached the critical value of 50°, we stopped the trajectory assuming that strongly twisted structures undergo fast and irreversible non-adiabatic transitions to the ground state. Figure 8 shows the evolution of the two populations (**A** and **B**) in the studied proteins. As one can see, in the EGFP-T65G mutant the chromophore eventually undergoes twisting in the course of excited-state dynamics. The twisting dynamics can be used to estimate the rate of the radiationless relaxation using first-order kinetics fit of **A**(t):

(2) $$A\left(t\right)=e^{-kt},$$

with radiationless (non-radiative) half-life τ_nr_:

(3) $$\tau_{nr}=\frac{ln\left[2\right]}{k}.$$

Fitting the decay of the planar population (shown in Figure 8) by first-order kinetics yields half-lives of 5.92 ns and 0.25 ns for EGFP and EGFP-T65G, respectively. In EYFP and EYFP-G65T the computed half-lives are 1.73 ns and 10.8 ns, respectively. These numbers roughly correspond to the excited-state decay via radiationless relaxation. As one can see, the T65G mutation in EGFP leads to a 23-fold drop in the non-radiative lifetime, which is in a semi-quantitative agreement with the 10-fold drop in FQY. The effect of the mutation of residue 65 in EYFP is slightly smaller, only 6-fold, which is qualitatively in agreement with 30% larger FQY in EYFP-G65T relative to EYFP. Addition of halide to EYFP leads to faster twisting by a factor of three, because halide binding upsets π-stacking of the chromophore with Tyr203 [19].

## 3. Discussion

Photophysical properties of the fluorescent proteins are determined by an interplay between chromophore’s intrinsic electronic structure, its interactions with the surrounding residues, and several competing excited-state processes. We begin by outlining the connection between the macroscopic observables (extinction coefficients, brightness, and photostability) with the microscopic properties of the chromophores. The extinction coefficient is proportional to the intrinsic brightness of the chromophore as characterized by the oscillator strength of the S_0_–S_1_ transition. Apparent excited-state lifetime (τ) is a result of the intrinsic fluorescence lifetime, τ_fl_, and various non-radiative decay channels (τ_nr_):

(4) $$\frac{1}{\tau }=\frac{1}{\tau_{fl}}\;+\frac{1}{\tau {\;}_{nr}}.$$

The non-radiative channels include radiationless relaxation and bleaching. However, given the small quantum yield of bleaching in typical fluorescent proteins (<10^−5^) [7], the second term in Equation (4) is dominated by the radiationless relaxation lifetime.

FQY is determined by the ratio of the radiative and radiationless lifetimes:

(5) $$FQY=\frac{k_{fl}}{k_{fl}+k_{nr}}=\frac{\tau_{nr}}{\;\tau_{fl}+\tau_{nr}}.$$

That is, for a given τ_fl_, FQY is larger when radiationless decay is slow (longer τ_nr_). Conversely, for a fixed τ_nr_, FQY is larger for systems with shorter radiative lifetime. Intrinsic radiative lifetime is related to the chromophore’s excitation energy and oscillator strength by Equation (1), i.e., larger oscillator strength leads to shorter radiative lifetimes (i.e., faster fluorescence rate). The photostability of the fluorophores depends on the ratio of excited-state lifetime and the rate of the bleaching process, i.e., within the first-order kinetics, the yield of bleaching Y_bl_ can be estimated as:

(6) $$Y_{bl}=\frac{\tau }{\tau_{bl}}$$

This means that for a given rate of the bleaching process (via photo-oxidation or other photochemical processes), the yield of bleached forms is smaller for systems with shorter apparent excited-state lifetimes. As photostability is inversely proportional to Y_bl_, the ratios of 1/Y_bl_ can be interpreted as relative photostabilities. Of course, bleaching rates can vary significantly among different proteins, because electron-transfer pathways and the rates are sensitive to mutations [19]. Because of the high cost of such calculations, the effects of mutations on the rates of electron transfer are not investigated in the present study.

Our simulations suggest that the principal effect of the T65G mutation is two-fold: (i) it increases the oscillator strength, leading to shorter fluorescence lifetimes; and (ii) it increases chromophore’s flexibility in the excited state, leading to faster radiationless relaxation. These two trends qualitatively explain all results from Table 1:

- EGFP-T65G has a larger extinction coefficient than EGFP because of its larger oscillator strength;
- Because of the faster radiationless relaxation, EGFP-T65G has lower FQY than EGFP, as per Equation (5); likewise, EYFP has lower FQY than EYFP-G65T;
- Having glycine in position 65 leads to faster radiationless relaxation (shorter half-life of **A**), thus suppressing the bleaching and leading to an increased photostability;
- Larger FQY in EYFP relative to EGFP-T65G arises due to the suppression of torsional motions by the π-stacking interactions, which is reflected in longer radiationless half-life.

**Figure 9:**
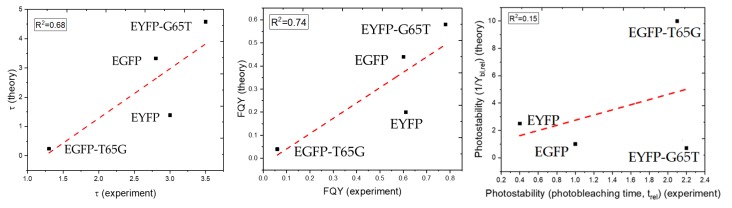
Correlation between theoretical and experimental apparent fluorescence lifetimes (**left**), FQY (**middle**), and the rate of bleaching (**right**).

To relate these calculations to the photophysical properties of the proteins, we collect the estimated half-lives due to radiationless relaxation and estimated radiative half-lives in Table 4. Figure 9 shows the comparisons between theory and experiment graphically.

We estimate FQY and the relative rates of bleaching using Equations (5) and (6). Although the computed FQY is not in quantitative agreement with the experimental values (which is not surprising, given that there are several approximations in the model), the differences between the mutants are described rather well: compare, for example, the ratio of FQY in EGFP-T65G and EGFP: 0.09 (computed) versus 0.1 (experimental). Likewise, the computed ratio of FQY in EYFP and EYFP-G65T is 0.34, to be compared with the experimental ratio of 0.78. The trend in the apparent fluorescence lifetime (τ) is also captured reasonably well: the computed relative lifetimes are 1:0.1:0.4:1.4, to be compared with the experimental ratios of 1:0.4:1.1:1.4 (estimated from the fluorescence lifetimes from Table 1).

Finally, the estimated bleaching yield (assuming the same rate of bleaching process) ratios are 1:0.1:0.4:1.4. The relative photostabilities can be estimated as the ratios of the inverse of Y_bl_: 1:10:2.5:0.71. The experimental macroscopic bleaching half-lives, which are inversely proportional to the rate of bleaching and can, therefore, be interpreted as relative photostabilities, are 1:2.1:0.4:2.2 (see Table 1, PBS). As anticipated above, the agreement here is worse, because the present study does not account for the variations in electron-transfer rates due to the mutations. The rate of bleaching is expected to vary between different systems, because the rate of electron transfer is sensitive to structural variations, especially between EGFP and EYFP [18]. In EYFP, the rate of bleaching is strongly affected by the halide binding, for example, the calculated electron-transfer rate to Tyr145 are five orders of magnitude faster in EYFP+Cl^−^ (k_EYFP+Cl-_:k_EYFP_ = 13300:1), which overshadows small variations in radiationless relaxation rates.

We conclude this section by considering the implications of the above findings for imaging applications. Faster radiationless relaxation of EGFP-T65G may result in the suppression of not only the bleaching but also other excited-state processes. This might be valuable from the practical point of view, for example, in the context of live-cell imaging where fluorophores with the decreased photoreactivity would potentially demonstrate such advantages as the decreased phototoxicity and fewer artifacts due to the redox-photoreactions with the intracellular compounds.

The high spectral sensitivity of EYFP-G65T to chloride (and probably to other anions of similar size) may open up new avenues for the design of FP-based molecular indicators, including those functioning in the lifetime domain. This is of special interest since EYFP and its circularly permuted variants have been utilized in several popular indicators [37,38,39].

## 4. Materials and Methods

The experimental measurements were carried out as follows. His-tagged proteins were expressed in *Escherichia coli* and purified by a metal-affinity resin. The resin beads with immobilized proteins were placed into phosphate buffer (PB, pH 7.4), or phosphate buffered saline (PBS, pH 7.4), or PB/PBS with 0.2 mM potassium ferricyanide as an oxidant and illuminated with strong blue light using a fluorescence microscope. Changes of fluorescence in green/yellow and red channels were monitored during illumination.

### 4.1. Spectroscopy and Fluorescence Brightness Evaluation

For absorbance and fluorescence excitation-emission spectra measurements, Cary 100 UV/VIS spectrophotometer and Cary Eclipse fluorescence spectrophotometer (Varian, Palo Alto, CA, USA) were used. Fluorescence brightness was evaluated as a product of molar extinction coefficient by quantum yield multiplication. Measurements on all native proteins were carried out in phosphate buffered saline (PBS, pH 7.4, Gibco, Paisley, UK). For molar extinction coefficient determination, we relied on measuring mature chromophore concentration [40]. EYFP and its mutants were alkali-denatured in 1 M NaOH. Under these conditions GFP-like chromophore is known to absorb at 447 nm (2.77 eV) with extinction coefficient of 44,000 M^−1^ cm^−1^. Based on the absorption of the native and alkali-denatured proteins, molar extinction coefficients for the native states were calculated. For determination of the quantum yield, the areas under fluorescence emission spectra of the mutants were compared with equally absorbing EYFP (quantum yield 0.61) and EGFP (quantum yield 0.60) [39].

### 4.2. Microscopy

For wide-field fluorescence microscopy, a Leica AF6000 LX imaging system with Photometrics CoolSNAP HQ CCD camera was used. Green and red fluorescence images were acquired using 63× 1.4NA oil-immersion objective and standard filter sets: GFP (excitation BP470/40, emission BP525/50) and TX2 (excitation BP560/40, emission BP645/75). Photobleaching and redding were monitored in time-lapse imaging in the green and red channels at low light intensity combined with exposures to blue light of maximum intensity (GFP filter set, light power density of 2–3 W/cm^2^). Images were acquired and quantified using Leica LAS AF software.

### 4.3. Protein Expression and Purification

EYFP and EGFP as well as EGFP-T65G and EYFP-G65T mutants were cloned into the pQE30 vector (Qiagen, Germantown, MD, USA) with a 6His tag at the N terminus, expressed in *E. coli* XL1 Blue strain (Invitrogen, Carlsbad, CA, USA) and purified using TALON metal-affinity resin (Clontech, Mountain View, CA, USA).

### 4.4. Site-Directed Mutagenesis

The EGFP-T65G and EYFP-G65T mutants were generated using overlap-extension PCR technique with the following oligonucleotide set containing the appropriate substitutions: forward 5′-ATGCGGATCCATGGTGAGCAAGGGCGAG-3′, reverse 5′-ATGCAAGCTTTTACTTGTACAGCTCGTC-3′ and forward 5′-ACCACCTTCACCTACGGCCTG-3′ and reverse 5′-CAGGCCGTAGGTGAAGGTGGT-3′ for EYFP G65T; forward 5′-ATGCGGATCCATGGTGAGCAAGGGCGAG-3′, reverse 5′-ATGCAAGCTTTTACTTGTACAGCTCGTC-3′ and forward 5′-ACCACCTTCGGCTACGGCCTG-3′, reverse 5′-CAGGCCGTAGCCGAAGGTGGT-3′ for EGFP-T65G. For bacterial expression, a PCR-amplified BamHI/HindIII fragment encoding an FP variant was cloned into the pQE30 vector (Qiagen).

### 4.5. Fluorescence Lifetime Imaging Microscopy of the Purified Proteins upon Single-Photon Excitation

Femtosecond laser pulses (80 MHz repetition rate, up to 100 fs, up to 25 nJ per pulse) were generated by a Ti:Sapphire oscillator (Tsunami, Spectra-Physics, Santa Clara, CA, USA) pumped by a green Nd:YVO4 CW laser (532 nm, Millennia Prime 6sJ, Spectra-Physics, Santa Clara, CA, USA) and frequency doubled in an LBO nonlinear crystal (Spectra-Physics, Santa Clara, CA, USA). Second harmonic laser beam was coupled to an inverted optical microscope Olympus IX71 by a Thorlabs FESH0750 dielectric filter mounted at 45° and then focused by objective lens (40 × 0.75NA, UPlanFLN, Olympus, Tokyo, Japan) on a sample, which was placed on a three-axis stage. The samples were prepared as droplets of the purified fluorescent proteins dissolved in phosphate buffered saline (PBS, pH 7.4, Gibco, Paisley, UK) applied onto a standard 24 × 24 mm cover glass (Heinz Herenz, Germany). The average laser power was tuned with a polarizing attenuator and further attenuated with a glass neutral filter. A typical laser power coupled to the microscope was about 3 μW. The central wavelength of the fundamental harmonic pulses was either 800 or 980 nm, and of the second harmonic pulses—400 or 490 nm, respectively. The SF10 prism compressor was used to compensate for the group velocity dispersion in the objective lens and other optical elements. Fluorescence was excited by one-photon absorption of femtosecond laser, passed back through the objective lens and laser coupling filter, was filtered by a long-pass dielectric filter (FELH0500, Thorlabs, Newton, NJ, USA), and then was directed to the input of Acton SP300i monochromator with two separate outputs. PI-MAX 2 CCD camera (Princeton Instruments, Trenton, NJ, USA) at the first output was employed for the fluorescence spectra registration. Photomultiplier tube of the time-correlated single photon counting system SPC-730 (Becker & Hickl GmbH, Berlin, Germany) at the second output detected the fluorescence decay kinetics in the 510–530 nm band. Fluorescence decay data were primarily acquired using SPCImage software (Becker & Hickl, Germany) and then exported in ASCII format and analyzed using Origin Pro 9 software (OriginLab, Northampton, MA, USA).

### 4.6. Computational Details

#### 4.6.1. Protonation State and Crystal Structures

Structures of EGFP and EYFP were taken from the protein data bank (PDB) with IDs: 2Y0G and 1F0B, respectively [11,15]. The protonation states of titratable residues were determined using PropKa software [27,28,29]. Particularly important are the protonation states of the residues around the chromophore [19]: Glu222 and His148. PropKa [28] predicts the glutamate to be neutral (GLUP 222) for both EGFP and EYFP. To validate this prediction, we carried out separate molecular dynamics simulations of EGFP and EYFP with protonated and deprotonated Glu222 and analyzed the key interatomic distances from the equilibrium trajectories. Direct comparison of these calculated distances with the crystal structures confirmed dominant population of the protonated Glu222 residue. This conclusion is also in accord with the prior experimental findings [13]. In EGFP, we visually inspected the local environment around the His148 residue and concluded that it exists in HSD (neutral, protonated at δN atom) form because of hydrogen bonding with the phenolate oxygen of the chromophore. Additional confirmation of HSD protonation state was obtained from similar His148-chromophore distances from equilibrium molecular dynamics simulation and the crystal structure. The resulting hydrogen-bond patterns around the chromophore are shown in Figure 4.

#### 4.6.2. Molecular Dynamics Setup

We used CHARMM27 force-field parameters for protein residues [41] and the ground-state anionic chromophores force-field parameters were obtained from [42]. Charged amino acids on the surface were locally neutralized by adding counter ions close (~4.5 Å) to them. Charged residues that do not form a salt bridge inside the protein barrel were also neutralized by adding appropriate counter-ions at the surface. This neutralization protocol resulted in the addition of 21 Na^+^ and 14 Cl^–^ in the EGFP, and 20 Na^+^ and 14 Cl^−^ in the EYFP. The proteins were solvated in water boxes producing a buffer of 15 Å with the box size of approximately 69 Å × 77 Å × 75 Å. The TIP3P [43] water model was used to describe external waters.

Molecular dynamics simulations were performed using these solvated neutralized model structures as follows:

1. Minimization for 2000 steps with 2 fs time step.
2. Equilibration of the solvent (keeping the protein frozen) for 500 ps with 1 fs time step.
3. Equilibration of the system for 2 ns (with 1 fs time step) with periodic boundary condition (PBC) under the isobaric–isothermal NPT ensemble.
4. Production run for 2 ns with 1 fs time step in an NPT ensemble.

Molecular dynamics simulations were performed with NAMD [44] in an NPT ensemble with Langevin dynamics. Pressure and temperature were kept at 1 atm and 298 K during the simulation.

#### 4.6.3. QM/MM Setup

We computed electronic properties (vertical excitation energies, oscillator strengths) using snapshots generated along equilibrium trajectories (production runs of molecular dynamics simulations) using a QM/MM scheme. The chromophore is included in the QM region and the rest of the system is treated as fixed MM point charges (see Figure 5) via electrostatic embedding. Hydrogen atoms were added at the QM/MM boundary to saturate the valencies. Point charges on the red and green atoms in Figure 5 were set to zero and the excess charge was redistributed over the rest of the atoms of the respective residues to avoid over-polarization of the QM atoms at QM/MM boundary.

Electronic structure calculations were performed at the ωB97X-D/aug-cc-pVDZ [45,46] level of theory. Benchmark results using different electronic structure methods are presented in the Supplementary Materials (Tables S1–S4, Figures S1–S3).

All quantum chemistry and QM/MM calculations were carried out using the Q-Chem electronic structure package [47].

## 5. Conclusions

In this contribution, we investigated the effect of residue in position 65 on the photophysical properties of EGFP and EYFP, with an emphasis on photostability and oxidative redding. We compared bleaching and redding kinetics in EGFP, EYFP, and their mutants with reciprocally substituted chromophore residues, EGFP-T65G and EYFP-G65T. Measurements showed that T65G mutation significantly increases EGFP photostability and inhibits its excited-state oxidation efficiency. Remarkably, while EYFP-G65T demonstrated highly increased spectral sensitivity to chloride, it is also able to undergo redding in the absence of chloride.

To shed light on the origin of the observed differences in photophysical behavior of the two seemingly very similar chromophores, TYG (EGFP and EYFP-G65T) and GYG (EYFP and EGFP-T65G), we carried out atomistic simulations of the four model systems.

The effect of the residue in position 65 can be explained by a simple kinetic model of the photocycle, which considers the competition between radiative and radiationless relaxation channels and photochemical bleaching. The atomistic simulations reveal that the main effect of the T65G mutation is the reduced excited-state lifetime of the GYG chromophore, resulting in its increased photostability. The effect of the residue in position 65 on the brightness and quantum yield is explained by an interplay between the radiative and radiationless relaxation channels.

Directed simulation- and structure-guided tuning of a relative significance of the radiative/radiationless processes can be a basis for the development of the new fluorescent proteins with pre-determined photostability and fluorescence lifetime optimized for application in the next-generation imaging techniques.

## Figures and Tables

**Table 1:** Fluorescent properties of EGFP, EYFP, and their mutants, EGFP-T65G and EYFP-G65T.

Fluorescent Protein	λex/λem, nm	EC, M^−1^cm^−1^	FQY	Relative Brightness, % *	Fluorescence Lifetime, ns **		Photobleaching, s ^#^				Redding Rate	
					PB	PBS	PB	PBS	PB + Ox	PBS + Ox	PB	PBS
**EGFP**	**489/509**	**55000**	**0.60**	100	n/d	2.8	n/d	80 ± 10	n/d	5 ± 2.5	n/d	strong
EGFP-T65G	488/508	70000	0.06	13	n/d	1.3	n/d	170 ± 25	n/d	85 ± 15	n/d	weak
EYFP	514/526	83400	0.61	154	3.18 ± 0.07	3.0 ± 0.08	21 ± 2	35 ± 2	3 ± 2	2 ± 1	no	moderate
EYFP-G65T	510/525	n/d	0.78	n/d	3.7 ± 0.06/4.0 ± 0.09	3.5 ± 0.07 and 0.5 ± 0.07/ 3.9 ± 0.08	25 ± 8	180 ± 8	10 ± 2	32 ± 7	moderate	moderate

n/d—not determined. * Relative brightness is calculated as a product of the molar extinction coefficient and the fluorescence quantum yield, and reported relative to the brightness of EGFP. For EGFP and EYFP, the absolute quantum yields are shown, for the mutants the quantum yields measured relative to the equally absorbing EGFP or EYFP are shown. ** For EGFP and EGFP-T65G literature lifetime values are shown [17]. For EYFP the single fluorescence lifetime value measured under 450 nm excitation is shown. For EYFP-G65T a pair of values measured under 400 and 490 nm excitation (value at 400/value at 490) is shown. In PBS, at 400 nm excitation EYFP-G65T showed fluorescence decay better fitted by bi-exponential function (τ_1_ = 3.5 ns, τ_2_ = 0.5 ns). ^#^ Photobleaching is reported as the bleaching half-time for each fluorescent protein, i.e., larger values correspond to the slower photobleaching rate and higher photostability.

**Table 2:** Average number of hydrogen bonds (and standard deviation) formed within 6 Å around the chromophore computed along the equilibrium ground-state trajectories. Distance and angle cutoffs were set to 3.2 Å and 20°, respectively. Deviation of the chromophore from planarity (Δ, in degrees) is also shown.

Protein/Chro	EGFP/ TYG	EGFP-T65G/ GYG	EYFP/ GYG	EYFP-G65T/ TYG	EYFP+Cl^–^/ GYG
Average No. of hbond	2.81	2.31	1.34	1.93	1.45
STD (hbond)	1.12	1.03	0.83	1.06	0.87
Δ	7.40	6.44	4.41	5.50	7.02
STD (Δ)	16.7	8.2	7.8	8.0	7.29

**Table 3:** Theoretical estimates of radiative lifetime for different mutants. Computed excitation energies and oscillator strengths are also shown. QM/MM absorption energies and oscillator strengths are averaged over 21 snapshots taken from ground-state equilibrium molecular dynamics simulations. τ_fl,rel_ values are relative lifetimes calculated with respect to τ_fl_ in EGFP.

Protein	E_ex_, eV (f_l_)		τ_fl_, ns			τ_fl,rel_	
	Gas phase	QM/MM	Gas phase (*n* = 1)	QM/MM (*n* = 1)	QM/MM (*n* = 1.6)	Gas phase	QM/MM
EGFP	3.101 (1.02)	3.081 (0.97)	29.50	31.24	7.63	1.00	1.00
EGFP-T65G	3.123 (1.05)	3.142 (1.04)	28.25	28.18	6.88	0.95	0.90
EYFP	3.123 (1.05)	3.097 (1.05)	28.25	28.71	7.02	0.95	0.92
EYFP-G65T	3.101 (1.02)	3.015 (0.98)	29.50	32.49	7.94	1.00	1.04
EYFP+Cl^–^	3.123 (1.05)	3.077 (1.07)	28.25	28.57	6.97	0.95	0.91

**Table 4:** Computed radiative and radiationless lifetimes of EGFP, EGFP-T65G, EYFP, EYFP-G65T, and EYFP+Cl^–^ (in parenthesis, the values relative to EGFP are shown) and estimated photophysical parameters.

Protein	τ_fl_, ns (τ_fl,rel_) ^a^	τ_nr_, ns (τ_nr,rel_)	τ, ns (τ_rel_) ^b^	FQY	Y_bl,_ rel ^c^
EGFP	7.63 (1.00)	5.92 (1.00)	3.33 (1.0)	0.44	1.0
EGFP-T65G	6.88 (0.90)	0.25 (0.04)	0.24 (0.1)	0.04	0.1
EYFP	7.02 (0.92)	1.73 (0.29)	1.39 (0.4)	0.20	0.4
EYFP-G65T	7.94 (1.04)	10.8 (1.82)	4.58 (1.4)	0.58	1.4
EYFP+Cl^–^	6.97 (0.91)	0.57 (0.10)	0.53 (0.2)	0.07	0.2

^(a)^ τ_fl_ estimated with Equation (1) and *n* = 1.6. ^(b)^ Estimated using Equation (4) ^(c)^ Yield of bleaching relative to EGFP using Equation (6) and assuming the same rate of bleaching in all proteins.

## Supplementary Materials

Supplementary materials can be found at https://www.mdpi.com/1422-0067/20/20/5229/s1.

## Abbreviations

avGFP	Aequorea victoria green fluorescent protein
EC	Extinction coefficient
EGFP	Enhanced green fluorescent protein
ET	Electron transfer
EYFP	Enhanced yellow fluorescent protein
FL	Fluorescence lifetime
FP	Fluorescent protein
FQY	Fluorescence quantum yield
GFP	Green fluorescent protein
GYG	Glycine-tyrosine-glycine
PES	Potential energy surface
PB	Phosphate buffer
PBS	Phosphate buffered saline
QY	Quantum yield
SYG	Serine-tyrosine-glycine
TYG	Threonine-tyrosine-glycine
QM/MM	Quantum mechanics/molecular mechanics
