# Supplementary material for: Influence of the First Chromophore-Forming Residue on Photobleaching and Oxidative Photoconversion of EGFP and EYFP
Source: Int J Mol Sci. 2019 Oct 22;20(20):5229. doi: 10.3390/ijms20205229 (PMC6829896; doi:10.3390/ijms20205229)
Supplement: Supplementary file 1 [file ijms-20-05229-s001.pdf]

International Journal of
Molecular Sciences

Supplemental information

Absorbance and fluorescence data normalization

Absorption and fluorescence excitation/emission spectra (fig.1) were normalized to the maximum mean value. Photobleaching curves (fig.2) were background-subtracted and normalized to the maximum values. EGFP and EGFP-T65G redding kinetics are shown as non-normalized and non-averaged background-subtracted curves (fig.3A). In the case of EYFP and EYFP-G65T redding representation, we had to take into account the fact that fluorescence of the yellow (non-converted) spectral form, routinely detected in GFP channel, has a spectral crosstalk with the red (RFP) channel, thus complicating correct red form appearance registration. Namely, redding-specific signal was masked by the "background" yellow fluorescence. To address this issue, we subtracted the background signal acquired in RFP channel considering its kinetics strictly proportional to that observed in GFP channel (according to the following equation RFPcorrected=RFPraw-(GFPraw*RFPzero/GFPzero), where raw is unprocessed value, zero – initial value).

The products of subtraction described above are represented either as maximum yellow signal-normalized and averaged curves (fig.3B) or as non-normalized single measurements (fig.3C).

Force field parameters for excited-state classical molecular dynamics simulations

To perform excited-state molecular dynamics, we reparameterized partial charges, key bond lengths, angles, and torsional angles, as well as the corresponding force constants. First, we optimized the structure of the isolated chromophore with ωB97x-D/aug-cc-pVDZ in its ground and the first excited state. We then computed the NBO charges [1] with the same functional/basis set and used the differences between ground and excited-state charges to calculate the partial charges in the excited state force-field by using the following equations

$$q_{ex-charmm} = q_{gs-charmm} + \Delta q_{NBO(ex-gs)}$$

$$where, \Delta q_{NBO(ex-gs)} = q_{NBO(ex)} - \Delta q_{NBO(gs)}$$

Bond lengths, angles, and dihedral angles were computed with the equations

$$E = k(b - b_0)^2$$

$$E = k(A - A_0)^2$$

Force constant (k) for excited state is computed as

$$k_{ex} = \frac{k_{ex}, theory}{k_{gs}, theory}\ k_{gs}$$

Where b_0 is equilibrium bond length and A_0 is equilibrium bond angle.

The force constants were computed by tweaking the parameters from the equilibrium value in ground and excited states by constructing the PES. The second derivative of the parabolic fit gives the force constant. We took the ratio of the computed force constant and multiplied that with those in ground state force constants:

$$E = k[1 + cos(n\varphi - \delta)],$$

where n is periodicity, δ is phase, φ = dihedral angle.

The most important parameter was the torsional angle φ. The PES scans show that the chromophore is planar in the ground state and twisted in the excited state. We then fitted the excited-state potential with a fitting potential with the calculated low force constant which enables the flip around φ.

Partial charges and other force field parameters are listed at force field section.

Fitting the potential for excited state PES (right) with respect to φ:

$$E = k[1 + cos(n\varphi - 180)], in\ ground\ state, n = 2$$

$$E = k[1 + cos(n\varphi - 180)], in\ excited\ state, n = 4$$

The major difference in the ground and excited state PES other than force constants is the change in periodicity (n) of the fitting potentials with much lower value of force constant for the torsional angle φ.

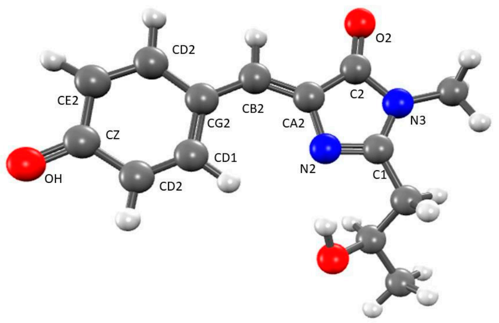

Figure S1. EGFP chromophore with atom types consistent with CHARMM 27 forcefield notations.

Table S1. Partial charges in Charmm27, and in the ground and excited states of the EGFP chromophore (ωB97X-D/aug-cc-pVDZ). The last column shows adjusted partial charges used in excited-state molecular dynamics.

Atom	Charmm27 (gs)	NBO (gs)	NBO (ex)	$\Delta q_{NBO(ex-gs)}$	Charmm 27 (ex)
C1	0.50	0.43	0.41	-0.02	0.48
N2	-0.60	-0.56	-0.64	-0.08	-0.68
N3	-0.57	-0.54	-0.54	0.00	-0.57
C2	0.57	0.66	0.61	-0.05	0.52
O2	-0.57	-0.75	-0.68	0.07	-0.50
CA2	0.10	-0.08	0.17	0.25	0.35
CB2	-0.14	-0.09	-0.47	-0.38	-0.52
HB2	0.21	0.25	0.25	0.00	0.21
CG2	-0.09	-0.21	0.06	0.27	0.18
CD1	-0.08	-0.17	-0.25	-0.08	-0.16
HD11	0.14	0.21	0.21	0.00	0.14
CD2	-0.08	-0.17	-0.25	-0.08	-0.16
HD21	0.14	0.24	0.24	0.00	0.14
CE1	-0.28	-0.31	-0.22	0.09	-0.19
HE11	0.10	0.21	0.21	0.00	0.10
CE2	-0.28	-0.31	-0.22	0.09	-0.19
HE21	0.10	0.23	0.23	0.00	0.10
CZ	0.45	0.46	0.39	-0.07	0.38
OH	-0.62	-0.72	-0.67	0.05	-0.57
CA3	-0.18	-0.41	-0.41	0.00	-0.18

HA31	0.09	0.26	0.26	0.00	0.09
HA32	0.09	0.20	0.20	0.00	0.09
C	0.51	(H)0.21	(H)0.21	0.00	0.51
O	-0.51	---	---	--	-0.51
N	-0.47	(H)0.24	(H)0.24	-0.00	-0.47
HN	0.31	---	---	--	0.31
CA	0.07	-0.51	-0.51	0.00	0.07
HA	0.09	0.24	0.24	0.00	0.09
CB1	0.14	0.09	0.10	0.01	0.15
HB1	0.09	0.20	0.20	0.00	0.09
OG1	-0.66	-0.77	-0.82	-0.05	-0.71
HG1	0.43	0.50	0.52	0.02	0.41
CG1	-0.27	-0.65	-0.65	0.00	-0.27
HG11	0.09	0.21	0.21	0.00	0.09
HG12	0.09	0.20	0.20	0.00	0.09
HG13	0.09	0.23	0.23	0.00	0.09

NOTE: Largest changes in partial charge happen on the methine bridge (marked by red).

Table S2. Bond lengths in Charmm27 forcefield and computed with ωB97X-D/aug-cc-pVDZ. The last column shows adjusted partial charges used in excited-state molecular dynamics.

Bond1	$K_{gs\text{-}charmm}$	$b_{0(gs\text{-}charmm)}$	$\Delta b_{0(computed)}$	$b_{0(ex\text{-}charmm)}$	$K_{gs\text{-}computed}$	$K_{ex\text{-}computed}$	$K_{ex\text{-}charmm}$
CG2Q-CB2Q	437	1.410	0.035	1.445	564.301	327.936	253.96
CB2Q-CA2Q	500	1.390	0.000	1.390	623.96	430.6	345.05

Table S3. Bond angles in Charmm27 forcefield and computed with ωB97X-D/aug-cc-pVDZ. The last column shows adjusted partial charges used in excited-state molecular dynamics.

Angle	$K_{gs\text{-}charmm}$	$A_{0(gs\text{-}charmm)}$	$\Delta A_{0(computed)}$	$A_{0(ex\text{-}charmm)}$	$K_{gs\text{-}computed}$	$K_{ex\text{-}computed}$	$K_{ex\text{-}charmm}$
CG2Q-CB2Q-CA2Q	130.0	133.2	-5.50	127.7	201.96	195.058	123.1

Table S4. Dihedral angles in Charmm27 forcefield and computed with ωB97x-D/aug-cc-pVDZ. The last column shows adjusted partial charges used in excited-state molecular dynamics.

Dihedral angle	$K_{gs\text{-}charmm}$	Periodicity (gs)	Periodicity (ex)	K_{gs}	K_{ex}	$K_{ex\text{-}charmm}$
φ	2.7	2	4	15.05	2.107	0.378
τ	3.9	2	4	14.992	1.479	0.39

Table S5. Computed excitation energy (eV), oscillator strength, and transition dipole moment (TDM, a.u.) at the ground-state optimized geometry of the isolated TYG and GYG chromophores.

Method	TYG			GYG		
	E_{ex} (eV)	f_1	TDM (a.u.)	E_{ex} (eV)	f_1	TDM (a.u.)
ωB97X-D/aug-cc-pVDZ (TDA)	3.367 (S_0-S_1)	1.425	4.156	3.387 (S_0-S_1)	1.467	4.204
ωB97X-D/aug-cc-pVDZ (RPA)	3.101 (S_0-S_1)	1.016	3.657	3.123 (S_0-S_1)	1.052	3.708
ωB97X-D/aug-cc-pVTZ (TDA)	3.363 (S_0-S_1)	1.420	4.151	3.382 (S_0-S_1)	1.460	4.198
ωB97X-D/aug-cc-pVTZ (RPA)	3.097 (S_0-S_1)	1.014	3.657	3.119 (S_0-S_1)	1.052	3.710
EOM-CCSD/aug-cc-pVDZ [2]	2.947 (S_0-S_1)	1.153	3.785	2.965 ((S_0-S_2)	1.188	3.839

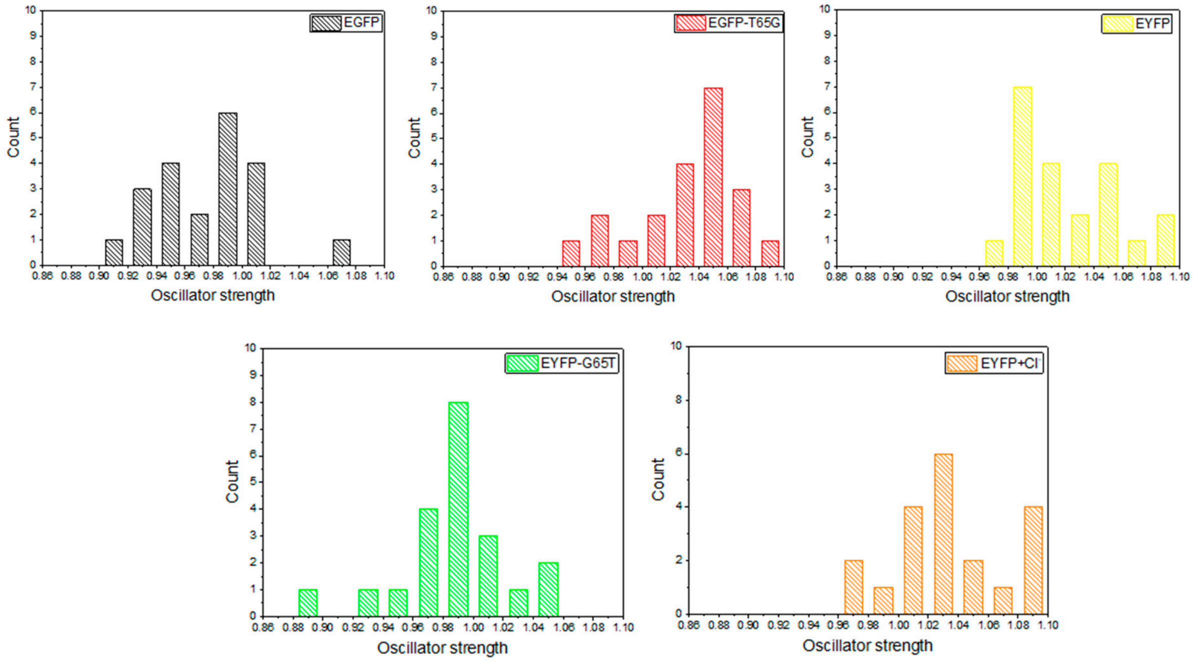

Figure S2. Distribution of oscillator strengths (ωB97x-D/aug-cc-pVDZ) computed for 21 QM/MM snapshots from the ground-state molecular dynamics.

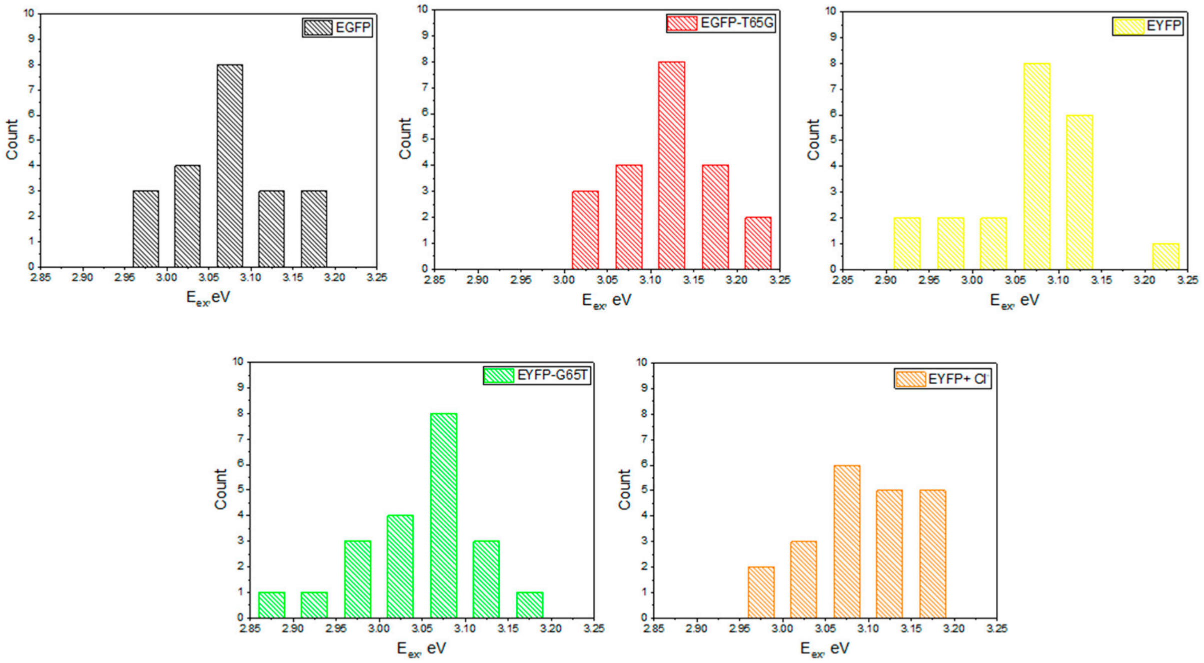

Figure S3. Distribution of vertical excitation energies (ωB97x-D/aug-cc-pVDZ) computed for 21 QM/MM snapshots from the ground-state molecular dynamics.

References

1. Glendening, E.D.; Landis, C.R.; and Weinhold, F. NBO 6.0: Natural bond orbital analysis program. *J. comput. chem.*, **2013**, *34*(16), 1429-1437.
2. Krylov, A. I. Equation-of-motion coupled-cluster methods for open-shell and electronically excited species: The hitchhiker's guide to Fock space Annu. Rev. Phys. Chem. **2008**, 59, 433–462.
